# GPCR in Adipose Tissue Function—Focus on Lipolysis

**DOI:** 10.3390/biomedicines11020588

**Published:** 2023-02-16

**Authors:** Davide Malfacini, Alexander Pfeifer

**Affiliations:** 1 Institute of Pharmacology and Toxicology, University Hospital, University of Bonn, 53127 Bonn, Germany; 2 Department of Pharmaceutical and Pharmacological Sciences, University of Padova, 35131 Padova, Italy

**Keywords:** Adipose tissue, WAT, BAT, lipolysis, G protein-coupled receptors (GPCRs)

## Abstract

Adipose tissue can be divided anatomically, histologically, and functionally into two major entities white and brown adipose tissues (WAT and BAT, respectively). WAT is the primary energy depot, storing most of the bioavailable triacylglycerol molecules of the body, whereas BAT is designed for dissipating energy in the form of heat, a process also known as non-shivering thermogenesis as a defense against a cold environment. Importantly, BAT-dependent energy dissipation directly correlates with cardiometabolic health and has been postulated as an intriguing target for anti-obesity therapies. In general, adipose tissue (AT) lipid content is defined by lipid uptake and lipogenesis on one side, and, on the other side, it is defined by the breakdown of lipids and the release of fatty acids by lipolysis. The equilibrium between lipogenesis and lipolysis is important for adipocyte and general metabolic homeostasis. Overloading adipocytes with lipids causes cell stress, leading to the recruitment of immune cells and adipose tissue inflammation, which can affect the whole organism (metaflammation). The most important consequence of energy and lipid overload is obesity and associated pathophysiologies, including insulin resistance, type 2 diabetes, and cardiovascular disease. The fate of lipolysis products (fatty acids and glycerol) largely differs between AT: WAT releases fatty acids into the blood to deliver energy to other tissues (e.g., muscle). Activation of BAT, instead, liberates fatty acids that are used within brown adipocyte mitochondria for thermogenesis. The enzymes involved in lipolysis are tightly regulated by the second messenger cyclic adenosine monophosphate (cAMP), which is activated or inhibited by G protein-coupled receptors (GPCRs) that interact with heterotrimeric G proteins (G proteins). Thus, GPCRs are the upstream regulators of the equilibrium between lipogenesis and lipolysis. Moreover, GPCRs are of special pharmacological interest because about one third of the approved drugs target GPCRs. Here, we will discuss the effects of some of most studied as well as “novel” GPCRs and their ligands. We will review different facets of in vitro, ex vivo, and in vivo studies, obtained with both pharmacological and genetic approaches. Finally, we will report some possible therapeutic strategies to treat obesity employing GPCRs as primary target.

## 1. Introduction

Adipose tissues store triacylglycerol in lipid droplets. WAT is the largest energy storage mechanism in the human body. In white adipocytes, the lipids are stored in a single big lipid drop (unilocular), while, in brown adipocytes, lipids are stored in multiple smaller droplets (multilocular) [1,2]. Another important difference is the mitochondrial content, which is very high in brown adipocytes, but low in white adipocytes [3]. In addition, brown adipocytes express a unique mitochondrial protein, the uncoupling protein-1 (UCP-1) [4,5]. UCP-1 is mainly responsible for releasing heat (thermogenesis) by uncoupling the respiratory chain [6,7]. Interestingly, brown-like adipocytes have been identified in human and murine WAT [2]. These adipocytes are induced by cold exposure or several drugs [8] and have been termed inducible brown, beige, or BRITE (brown-in-white) cells; together with “classical” brown adipocytes, they form the thermogenic adipose tissue.

**Figure 1:**
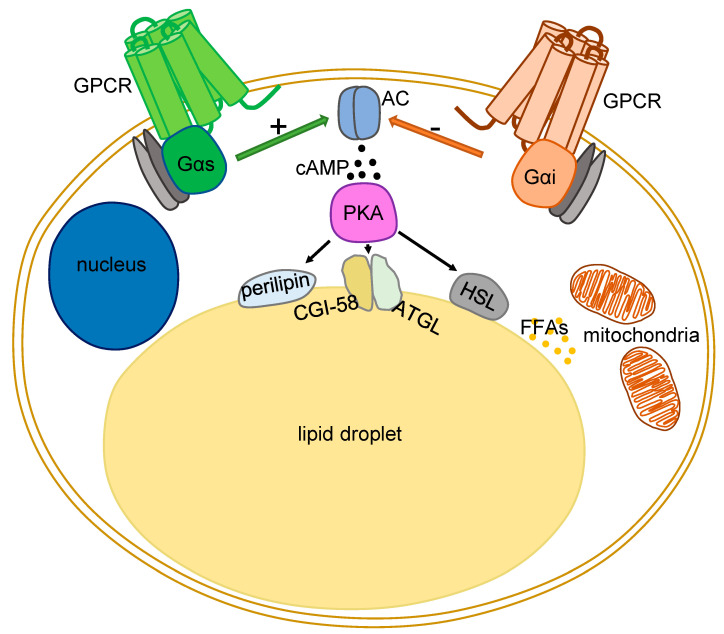
GPCR-regulated signaling events in adipocytes. Gs-coupled GPCR stimulates adenylate cyclase (AC) to convert adenosine triphosphate (ATP) to 3′,5′-cyclic AMP (cAMP) and pyrophosphate. Protein kinase A (PKA), in the presence of cAMP, phosphorylates perilipin and hormone-sensitive lipase (HSL). p-perilipin facilitates lipolysis mediated by other lipolytic enzymes while HSL hydrolyzes triacylglycerols and diacylglycerols, thus liberating free fatty acids (FFAs) used in the mitochondria or released in the extracellular matrix. CGI-58 and ATGL are involved in the early phases of the lipolytic process. While Gi-coupled GPCRs, on the contrary, inhibit the activity of AC, hindering lipolysis.

Three enzymes are mainly responsible for the catalysis of triacylglycerol into FFAs and glycerol: adipose triglyceride lipase (ATGL), hormone-sensitive lipase (HSL), and monoglyceride lipase (MGL) (Figure 1). The central regulator of lipolysis in adipocytes is the second messenger cAMP, which is produced from adenosine triphosphate (ATP) by a family of enzymes called adenylate cyclases (ACs). Nine out of ten AC isoforms are located in the plasma membrane, while one is found in the cytoplasm (soluble AC, sAC, AC10) [9]. Transmembrane ACs are modulated via several mechanisms: the best studied ones are the heterotrimeric G proteins (G proteins), composed of Gα and Gβγ subunits. Activation of G protein-coupled receptors (GPCRs) facilitates the GDP/GTP exchange in the Gα subunits of heterotrimeric G proteins (G proteins). Gα subunits can either stimulate or inhibit the levels of cAMP and are, therefore, named stimulatory (Gs) or inhibitory (Gi). 

The level of cAMP is tightly controlled not only by the production (i.e., ACs), but also by the breakdown of cAMP by phosphodiesterases (PDEs). Based on their specificity for the cyclic nucleotides cAMP and/or cGMP, the PDEs are subdivided into three major groups: PDE4, 7, and 8 specifically catalyze the hydrolysis of cAMP, whereas PDE5, 6, and 9 are cGMP specific, and PDE1, 2, 3, 10, and 11 hydrolyze both cAMP and cGMP [10]. Cell subtype expression and cellular subdomain localization of ACs and PDEs, together with specific buffering mechanisms, cause cAMP concentration to be tightly controlled at the nanometric scale [11], thus allowing compartmentalization and specificity of cAMP signaling [12,13]. 

The major receptor/mediator of cAMP effects in adipocytes is the cAMP-activated protein kinase (PKA). PKA in its inactive form is a tetramer consisting of two regulatory subunits and two catalytic subunits [14]. Binding of cAMP activates PKA, and the PKA catalytic subunit then phosphorylates several targets in the cytoplasm (e.g., PDEs, GSK3 [15,16,17]), at organelles (e.g., lipid droplets, mitochondria [18]), and in the nucleus [19,20] (e.g., the nuclear cAMP-response element-binding (CREB) protein and other transcription factors, which in turn regulated adipogenesis) [21]. 

Lipolytic enzymes are an important target for cAMP in adipocytes [22,23,24]. PKA phosphorylates perilipin A and hormone-sensitive lipase on lipid droplets and promotes lipolysis [25]. Lipid droplets are highly dynamic organelles playing a key role in the regulation of intracellular lipid storage and lipid metabolism, and they are surrounded by phospholipids and important regulating proteins [26]. Phosphorylation of perilipin A promotes the release of comparative gene identification (CGI)-58, a co-activator of ATGL [27] (Figure 1).

Importantly, insulin and Gi-coupled GPCRs can diminish the lipolytic rate. In contrast, Gs-coupled GPCRs, such as beta-adrenergic receptors, stimulate lipolysis. A huge number of GPCRs expressed by WAT and BAT have been identified [28]. However, the pathophysiological function has been unraveled for only a fraction of these GPCRs. 

## 2. GPCR Signaling 

GPCRs are seven-transmembrane spanning proteins communicating a plethora of extracellular signals over the cell membrane into the cell. GPCRs form the largest human membrane protein family, including approximately 800 members, of which about half are non-olfactory receptors. Importantly, GPCRs and their ligands are the target of approximately one third of all marketed drugs [29]. GPCRs are capable of sensing a wide spectrum of stimuli from odorants to photons, to ions, to metabolites and drugs. They are classified into five main families, named glutamate, rhodopsin, adhesion, frizzled/taste2, and secretin types of GPCRs (GRAFS classification) [30], of which the rhodopsin family is the most studied, and this includes most of the pharmacologically targeted GPCRs, such as those for catecholamines. 

Among the plethora of physiological processes controlled by GPCR signaling, the regulation of serum glucose levels (e.g., by activation of the pancreas islets glucagon receptor [31,32,33]) and lipolysis [34,35] are highly relevant for energy homeostasis. With this review, we provide an overview of GPCRs as potential pharmacological targets in adipose tissues. We primarily focus on lipolysis control and the regulation of lipid content and breakdown.

GPCR signaling is mediated primarily through G proteins [36]. Concerning the activity of G proteins in adipocyte function per se, pivotal experiments in mice showed that enhancing Gs signaling (via application of the cholera toxin, CTX) prevents age-associated obesity and inflammation [37]. Treatment of white adipocytes with CTX induces increased lipolysis [38]. Interestingly, similar results were obtained by blocking Gi signals via pertussis toxin (PTX) treatment [39,40]. The effects of Gs-coupled receptors in adipocytes are by-and-large mediated via cAMP pathway, whereas Gq effects are mediated by activation of the phospholipase C beta (PLC-β), which in turn hydrolyzes phosphatidylinositol 4,5-bisphosphate (PIP2) to diacyl glycerol (DAG) and inositol trisphosphate (IP3). DAG activates protein kinase C (PKC), while IP3 induces the release of intracellularly stored calcium. Intriguingly, pharmacological (i.e., FR900359) and genetic inhibition of Gq signaling has been shown to enhance differentiation of human and murine brown adipocytes, while activation of Gq abrogates brown adipogenesis [28]. Furthermore, recent studies revealed that Gq signaling inhibits lipolysis and stimulates glucose uptake in an insulin-independent fashion in both human and mouse white adipocytes, thereby improving lipid homeostasis in obese mice [41]. Overall, Gs- and Gi-coupled GPCRs are pivotal regulators of adipocyte lipid content (Figure 1).

## 3. “Classical” Activators of Lipolysis—Norepinephrine (NE) and Beta Receptors

The sympathetic nervous system (SNS) innervates AT and plays a key role in activating lipolysis, thereby promoting the release of energy in the form of FFAs or heat from the WAT and BAT, respectively. The most studied SNS transmitter at the neuronal-adipocyte interface is the catecholamine NE, as well as the three beta-adrenergic (beta 1-, beta 2-, and beta 3-adrenergic) receptors. Moreover, seminal work investigated the role of alpha adrenergic receptors role in lipolysis [42,43]. In particular, the selective pharmacological stimulation of the alpha 2 receptor in human white adipocytes inhibits lipolysis. Although, knockout mice lacking beta 1, beta 2, or beta 3 did not show obesity and/or major metabolic alterations [44,45,46,47]. Bachman and coworkers [48] showed that mice lacking all three adrenergic receptors (beta-less) have increased weight gain on a standard chow diet, and they exhibited a worsened obese phenotype when fed a high-fat [49]. Moreover, beta-less mice showed increased leptin levels, and their BAT was transformed to white fat (“whitened” BAT, with unilocular droplets). This phenotype of the beta-less mice was similar to denervated BAT [50] or catecholamine-deficient [51] mice. Oxygen consumption is a measure of energy expenditure [52,53], and it is lower in beta-less than in wild-type mice, underlining the importance of beta-adrenergic signaling for BAT and energy homeostasis. In beta-less mice, release of glycerol and non-esterified fatty acid from white adipocytes—both parameters proportional to lipolysis rate—were largely diminished in the presence of increasing doses of NE and isoproterenol, albeit not completely depleted. 

The beta 3 receptors have received a lot of attention in the context of adipocytes: beta 3 stimulation in mice determines an increased blood level of lipids more than three-fold [47]. This supports the growing body of preclinical evidence regarding the use of selective beta 3 agonists to activate BAT and energy expenditure. Although human studies show a similar picture [54], recent data indicate that beta 2 adrenergic receptors might be the predominant receptor major role in human BAT lipolysis [55,56,57,58]. Nevertheless, beta 3 adrenergic receptor signaling appears to be necessary for maximal brown and beige adipocyte lipolysis and thermogenesis [58]. Overall, a broader understanding of the different pharmacological properties of all three beta adrenergic receptors in humans and the polypharmacology of available and novel ligands, together with GPCR compartmentalization [59], could help to account for this phenomenon. 

## 4. Alternatives to NE—Adenosine Regulates Lipolysis

NE is not the sole trigger of lipolysis via activation of GPCRs [60]: the nucleoside adenosine and its break-down product inosine, which also plays a pivotal role, especially in brown adipocytes [61,62]. Adenosine is a building block of the genetic code, a central part of ATP—a key component of cellular energy homeostasis—and in the form of cAMP, of signal transduction [63,64]. Moreover, extracellular purine nucleosides modulate many physiological processes by interacting with P1 purinergic GPCRs—A1R, A2AR, A2BR, and A3R. Stimulation of these four GPCRs can elicit a wide plethora of effects, and this is also because of their different G protein coupling: Gi/Go for A1R/A3R and Gs for A2AR/A2BR [65]. Very early experiments conducted with isolated rat white adipocytes indicated adenosine release in the medium, and introduction of the adenosine degrading enzyme (adenosine deaminase) increased basal lipolysis [66]. Thus, this indicates a general inhibition of lipolysis caused by adenosine. Similarly, adenosine inhibited lipolysis in white hamster adipose tissue through activation of the Gi-coupled A1R [62]. Moreover, inhibition of lipolysis was shown in brown adipocytes from hamsters and rats. In contrast, lipolysis was induced in murine brown adipocytes by the addition of adenosine. These opposing effects of adenosine in AT of distinct species can be explained by different expression patterns of adenosine receptors (coupling to both lipolysis inhibitory and stimulatory G proteins) in different species [62]. Adenosine receptors are also differently expressed in different types of adipocytes, which could explain the much lower (144-fold) potency of adenosine in white versus brown adipocytes [62]. Blockade of the A1R (Gi-coupled) either pharmacologically or genetically shifted the concentration response curves for Adenosine (Ado) to the left in white adipocytes [62]. In contrast, blocking A2AR and A2BR completely abolished the effects of Ado on lipolysis in brown adipocytes [62]. In vivo studies further substantiated the importance of A2 receptors for BAT and energy homeostasis: Injection of an A2A agonist strongly increased whole-body oxygen consumption, reaching 70% of the maximal effect induced by NE without altering locomotor activity. In contrast, indirect calorimetry of cold-exposed adult A2A knock out mice revealed a 30% reduced BAT activity [62]. Moreover, adenosine and A2A agonist (PSB-0777)-induced respiration and lipolysis were blunted in the absence of A2A receptors [62]. Importantly, Ado stimulated lipolysis in primary human brown adipocytes and a human white/beige cell line (hMADS) [62]. Since A2 receptors have a predominant role in thermogenic fat physiology as compared to white fat [62,67], they might have a very high potential as drug target for body weight control.

Very recently, the composition of metabolites released by apoptotic BAT has been clarified: high concentrations of purine metabolites were detected [61]. Among these metabolites, inosine was the most highly up-regulated extracellular purine [61]. Interestingly, inosine stimulates energy expenditure in brown adipocytes by inducing cAMP production and PKA signaling. Mice treated with inosine exhibited increased BAT-dependent energy expenditure and ‘browning’ of WAT [61]. These effects of inosine are diminished by A2A and A2B receptors antagonists [61].

Finally, since purines are released upon NE-induced activation of brown/beige adipocytes [61], these could be viewed as two cooperating transmitter systems. 

## 5. Dopamine and Serotonin Receptors

Dopamine and serotonin receptors function on metabolism was mainly studied in the central nervous system. However, their direct role in adipose tissue lipolysis is still an object of debate. SKF38393 and bromocriptine, D1 (Gs) and D2 (Gi) receptors agonists, respectively, have antilipolytic actions on the adipose tissue in obese mice. Similarly, D1 and D2 agonism inhibited lipolysis in isolated murine adipocytes [68]. In humans, D2 agonists acutely ameliorated metabolic parameters, in particular, mean 24-h blood glucose and insulin were significantly reduced. In addition, D2 agonism increased oxygen consumption, resting energy expenditure, and diminished systolic blood pressure, together with an increase in FFAs 24 hr after treatment, suggesting that lipolysis was stimulated [69]. 

Serotonin and selective agonism of the 5-HT2A receptor (Gq-coupled) suppressed lipolysis in primary rat adipocytes [70]. On the other hand, inhibition of serotonin synthesis led to lipogenesis inhibition in gonadal WAT, stimulation of browning in subcutaneous WAT and enhanced thermogenesis in BAT. Serotonin effects on WAT and BAT seemed mainly due to the involvement of cation channels [71]; however, because of the high expression of serotonin sensitive GPCRs in adipocytes [28], further studies are needed to clarify their role in adipose tissues.

## 6. Free Fatty Acid Receptors (FFARs)

FFAs can bind specific GPCRs at the cell membrane, as well as intracellular receptors such as fatty acid binding proteins (FABPs) and peroxisome proliferator activated receptors (PPARs). FFAs are synthesized in a multistep process. Upon synthesis completion, FFAs generally bind to glycerol (three fatty acids bind to one glycerol) to form triglycerides. BAT and WAT, in their lipolytic processes, liberate FFAs in the cytoplasmic environment. Outside the cell, FFAs circulate bound to albumin and represent an energy source—in a process called beta-oxidation—for every mitochondrion containing cells. 

Seminal results with non-esterified FFAs (NEFAs) showed an inhibition of lipolysis [72,73]; interestingly, experiments carried out with synthetic derivatives indicated that this occurs in a GPCR-dependent manner and independently from intracellular activities alteration [74]. 

According to The Guide to Pharmacology [75], FFA-regulated GPCRs can be classified as follows: long-chain saturated and unsaturated fatty acids (including C14.0 (myristic acid), C16:0 (palmitic acid), C18:1 (oleic acid), C18:2 (linoleic acid), C18:3, (α-linolenic acid), C20:4 (arachidonic acid), C20:5,n-3 (EPA), and C22:6,n-3 (docosahexaenoic acid)) activate FFAR1 (Gs > Gi/o) and FFAR4 (Gq/11), while short chain fatty acids (C2-C5 (acetic to pentanoic acid)) activate FFAR2 (Gq/11 > Gi/o) and FFAR3 (Gi/o). 

Among GPCRs sensing FFAs, the FFAR4/GPR120 is abundantly expressed in white and brown adipocytes and adipose tissues, while it is much lower expressed in preadipocytes [76]. FFAR4 transcript levels were abundant in samples from insulin-resistant and diabetic mice (inguinal and gonadal WAT, and BAT), while in obese mice, FFAR4 expression was downregulated, thus indicating a complex regulation of FFAR4 expression in adipocytes’ surface [76]. Mice on a high-fat diet display higher expression of FFAR4, and in the genetic absence of FFAR4, an obese phenotype accompanied by inflamed adipose tissue and fatty liver was observed [77]. Conversely, human adipose tissues exhibited a higher expression of FFAR4 in lean rather than obese subjects [78]. In addition, cold exposure increased the abundance of FFAR4 in murine BAT [79]. Interestingly, in humans, the polymorphism R270H in at codon 270 (FFAR4, p.R270H, rs116454156) is described as a risk factor for obesity [80,81,82,83]. The R270H mutation leads to a diminished amplitude of FFAR4 signals [80]. Treatment of adipocytes with agonists TUG-891 (selective for the FFAR4) and GW9508 (selective for FFAR1/GPR40) induce deoxyglucose uptake [84,85], thus indicating a promising role to counteract obesity and insulin resistance. In vivo data employing a FFAR1/4 dual agonist showed improved hepatic insulin sensitivity. Activation of FFAR4 and Gq signaling with TUG-891 was reported to induce mitochondrial respiration [86]. However, the selectivity of TUG-891 at the human FFAR4 over FFAR1 receptors is not as high as for the murine receptors: FFAR1 is coupling to Gs and the observed TUG-891 effects might be mediated by FFAR1 activation [87]. In addition, administration of Cpd B a FFAR4 selective agonist, showed in rats decrease FFAs in blood without altering insulin levels. Cpd B dose-dependently suppressed white rat adipocytes lipolysis, while FFAR4 knockout mice did not show any effect of Cpd B on lipolysis [88]. Finally, Omega-3 fatty acids elicit their effects on FFAR4 localized in the primary cilium, a regulating organelle whose importance is an object of intense debate [89]. The variation of FFAR4 subcellular localization could account to FFAR4 signaling and function shift.

## 7. Endocannabinoid System and Receptors

WAT and BAT are capable of synthesizing endocannabinoids [90,91,92,93]. The 2-arachidonoylglycerol (2-AG) and anandamide (n-arachidonoylethanolamine, AEA) are endocannabinoids and endogenous ligands for the CB1 and CB2 receptors. Both GPCRs are coupled to Gi, thereby resulting in reduced lipolysis [94]. Interestingly, there is a link between the sympathetic nervous system and adipocyte crosstalk: the endogenous release of endocannabinoids seems to inhibit NE release because of their activation of presynaptic CB1 receptors of sympathetic neurons, thereby inhibiting lipolysis [95]. In addition, CB1 stimulation fostered lipogenesis [95], while the incubation of adipocytes or adipocyte-related cell lines with CB1 inverse agonists—ligands capable of decreasing the constitutive activity of GPCRs—stimulated lipolysis [96]. Moreover, the application of CB1 antagonists in vivo increased the levels of adiponectin with consequently increased insulin sensitivity [97]. 

One of the most promising approaches for CB1 targeting in the context of obesity was the CB1 inverse agonist rimonabant (SR141716, Acomplia) [98]. Rimonabant was developed as an anorectic antiobesity drug [99], but because of serious psychiatric problems, including suicide, was withdrawn from the market. Part of the antiobesity effects were due to CNS and part to targeting AT, because of a rimonabant-induced elevation in BAT temperature and decrease in body weight, which were attenuated following sympathetic denervation [100]. Moreover, application of rimonabant elicited an increase in oxygen consumption and glycerol release in the brown adipocyte cell line T37i [101]. 

Obesity increases CB2 expression in WAT [102], especially within the macrophage-enriched stromal vascular fraction. Intriguingly, a correlation between CB2 genetic variants and body mass has been reported [103]. Nevertheless, more studies are needed to elucidate the function of CB1 and CB2 receptors in the adipose tissues [93].

GPR55 and GPR18 were identified as putative cannabinoid receptors. GPR55 couples to G13 and Gq [104,105], and its transcripts are up-regulated in the visceral adipose tissue of obese compared to non-obese humans [106]. Ex vivo treatment of visceral and subcutaneous human fat with lysophosphatidylinositol, a GPR55 endogenous candidate ligand, are linked to calcium fluxes and upregulation of lipogenic genes [106]. GPR18 is also expressed in brown adipocytes, couples to Gi and Gq [107,108], and its putative endogenous ligand is N-arachidonylglycine [107].

Taken together, cannabinoid receptors are still object of intense study in the context of obesity. Rimonabant showed the potential and the hurdle (serious psychiatric problem) of targeting the cannabinoid system in the CNS. However, more specific experimental approaches, including adipose tissue-specific knock-out, are still needed to fully understand how CB1, CB2, GPR55, and GPR18 can orchestrate endocannabinoid response in metabolism, adipose tissue, and lipolysis.

## 8. Steroid- and Oxysterol Sensing GPCRs 

The benefit of estradiol administration in post-menopausal obesity was often linked to nuclear estrogen (alpha and beta) receptors; however, a recent report identified the G protein-coupled estrogen receptor (GPER) as a possible candidate for treatment of metabolic disorders associated with menopause [109]. G1, a GPER selective agonist, prevented body weight gain in diet induced obesity in mice where menopause was mimicked [109]. The effects were accompanied by improved plasma lipid profiles because of increased BAT functionality (mitochondrial gene expression and cellular respiration). G1 is also involved in mitochondrial biogenesis in WAT [110].

Another important steroid hormone responsive GPCR is GPRC6A. GPRC6A has a broad binding profile, being activated by basic amino acids [111], bivalent cations [112], uncarboxylated osteocalcin [112], and steroids [113]. Mice lacking GPRC6A in an adipocyte-specific (Fabp4/Ap2-Cre) manner showed adipocyte hypertrophy accompanied by impairment of lipolysis-related genes (ATGL and HSL) [114]. These mice did not lose weight as much as the wild-type upon fasting [114]. Moreover, they did not tolerate cold exposure as well as wild-type mice and showed limited release of NEFA upon isoproterenol administration; altogether, these data indicate that GPRC6A regulates lipolysis [114]. Wild-type epididymal WAT samples treated ex vivo with GPRC6A agonists displayed higher adipose triglyceride lipase levels compared to the knockout. 3T3-L1 cells treated with GPRC6A agonists showed increasing levels of intracellular cAMP, which should induce lipolysis, which was abolished by ectopic expression of small interference RNA against GPRC6A [115]. 

GPR183/EBI2 is among the most abundant inhibitory GPCRs expressed in BAT. 7α,25-dihydroxycholesterol—the endogenous ligand for EBI2—decreased BAT-mediated energy expenditure in mice [116]. Mice lacking EBI2 deficient for EBI2 show increased energy dissipation in response to cold [116]. EBI2 stimulation via 7α,25-dihydroxycholesterol decreased lipolysis effects mediated by NE in murine brown adipocytes and hMADS but not in white adipocytes; importantly, the effects were reverted by the use of a selective antagonist [116].

Collectively, steroids and their derivatives (e.g., oxysterols) are receiving greater attention for their capacity of regulating metabolism and adipose tissue function. Together with nuclear receptors activated by steroids [117], these GPCRs described above are crucial for adipose tissue regulation. 

## 9. Peptides GPCRs and Lipolysis

An amount of 118 out of 826 human GPCRs recognize endogenous peptide or protein ligands [118], with functions in the most diverse areas of life science. Here, we will focus on some examples relevant for adipocyte lipolysis.

### 9.1. Endothelin Receptors—ETA and ETB Receptors

The role of endothelin receptors (ETA and ETB receptors) in adipose tissues and obesity has been the object of intense studies. ETA receptor is mainly coupling to Gq in BAT [28], while ETB is coupling to different G proteins (Gs, Gi, Gq). ETA receptor expression was higher in obese than non-obese subjects in subcutaneous fat, while ETB receptor expression was unaltered [119]. Increasing concentrations of endothelin-1 (ET-1) showed no acute effect on lipolysis in freshly isolated human adipocytes [119]. However, primary cultures of human adipocytes showed a concentration-dependent effect of ET-1 on lipolysis evident only after longer incubation time (6 and 24 h) [119]. The effect could be mimicked by a selective agonist of the ETA (ET-1 [1,2,3,4,5,6,7,8,9,10,11,12,13,14,15,16,17,18,19,20,21,22,23,24,25,26,27,28,29,30,31]) receptor but not with BQ3020 a selective ETB receptor agonist [119]. In addition, ET-1 induced lipolysis in rat adipocytes and 3T3-L1 cells; however, this is conducted in a cAMP-independent manner [120,121]. Further studies are needed to clarify how the coupling of ETA receptor is capable of eliciting lipolysis in a possibly Gs independent manner or whether signaling promiscuity can account for these effects.

### 9.2. Chemerin

Chemerin is an 18 kDa protein secreted by adipose tissue (adipokine) known to modulate the immune system [122,123], and it binds the CMKLR1/ChemR23, a GPCR highly expressed in human subcutaneous adipose tissue [124]. Mice lacking CMKLR1 displayed mild obesity, without alterations in the differentiation of adipocytes [125]. Administration of chemerin in 3T3-L1 cells induced a significant increase in lipolysis [126], and chemerin gene downregulation depressed lipolysis [127]. However, high concentrations of chemerin counteracted cAMP-mediated lipolysis (isoproterenol and 3-isobutyl-1-methylxanthine (IBMX)), while activating ERK1/2 pathway, pointing to a more complex mechanism of action for chemerin in adipocytes. However, the specificity of the aforementioned effects needs to be further verified.

### 9.3. Apelin

Apelin gene encodes for a 77 amino acid preproprotein (pre-apelin) containing multiple enzyme cleavage sites leading to several bioactive peptides, i.e., apelin-36, apelin-17, apelin-13, and apelin-12. Apelin is expressed, together with the apelin receptor (a Gi-coupled GPCR), in the murine and human white adipose tissue [128]. This feature, secretion of apelin and presence on the surface of its GPCR, indicates autocrine mechanism of signaling [129]. However, few reports are addressing the role of the apelin receptor in adipose tissue, and lipolysis experiments have been performed, to the best of our knowledge, only in 3T3-L1 cells [130]. In these experiments apelin dose-dependently prevented adipogenesis and increased the size of lipid droplets, thus suggesting an inhibitory role on lipolysis [130], in line with its G protein coupling. Another report more directly identified the role of apelin in preventing beta-adrenergic stimulated lipolysis [131]. Taken together, results on the apelin receptor suggest to thoroughly investigate the potential of use of antagonists to induce lipolysis. In addition, inverse agonists might be of interest given the fact that a constitutive active form of the apelin receptor has been described [132].

### 9.4. Calcitonin Receptors

The calcitonin receptor functions as receptor complex with receptor activity modifying proteins (RAMP1, RAMP2, and RAMP3, respectively) [133]. Adrenomedullin, a multifunctional regulatory peptide that is produced and secreted by various types of cells, inhibited lipolysis [134] by nitric oxide (NO)-dependent mechanism (as shown in 3T3-L1 cells). Intriguingly, calcitonin signaling might play a role in sensory innervation of adipose tissues and neural-adipose network feedback to the brain can in turn lead to an increase in CNS-induced peripheral lipolysis [135,136,137]. Sympathetic denervation experiments showed that calcitonin gene related peptide (CGRP) immunoreactivity increased [138,139]. Importantly, ablation of the calcitonin receptor in mice resulted in impaired glucose tolerance and adipose tissue inflammation. In addition, calcitonin receptor-deficient mice exhibited dyslipidemia and elevated high-density lipoprotein levels [140]. This evidence supports the importance of the role of calcitonin receptor for adipose tissue function and lipolysis.

The signaling of calcitonin receptors is altered based on which GPCR complex is formed, therefore, calcitonin signaling in adipose tissues is complex and still an object of ongoing research. 

### 9.5. Neuropeptides

Neuropeptides sensing GPCRs are expressed not only in the CNS, but also in peripheral tissues, including the adipose tissues. As a thumb rule, central orexigenic neuropeptides have antilipolytic properties, conversely anorectic neuropeptides have lipolytic actions when expressed in adipocytes. For instance, in 3T3-L1 cells, the non-selective melanocortin receptor agonist MTII elicited a robust increase in lipolysis and glycerol release [141]. Among the melanocortin receptors, lipolysis is mediated by the stimulation of the MC2 and MC5 receptors (both Gs-coupled) [142]. Neuropeptide Y (NPY) decreased the basal free fatty acid release, while alpha-melanocyte-stimulating hormone (α-MSH) did induce free fatty acids release in murine white adipocytes [141]. In isolated human white adipocytes, NPY and peptide YY (PYY) displayed a robust inhibition of the lipolytic effect induced by adenosine deaminase. NPY effect was concentration-dependently, counteracted by the application of the two antagonists SR120819A and BIBP3226, thus indicating a major involvement of the NPY1 receptor (Gi-coupled) in the lipolysis regulation in human white adipocytes [143]. In addition, the NPY1 receptor has recently been the object of intensive investigation. Work by Yan and co-workers showed how peripheral NPY receptor is strongly involved in obesity development [144]. In fact, pharmacologic (i.e., BIBO3304) or genetic inhibition of peripheral NPY1 receptor prevents the development of high-fat diet induced obesity mainly in a thermogenic manner [144].

Neuropeptide FF (NPFF) is reported to activate two GPCRs, GPR74, and GPR147. Intriguingly, an ATAG haplotype of GPR74 was described to be associated with leanness and increased lipolysis in vivo (plasma glycerol corrected for body fat) and in vitro ([145]. Small interference RNA against GPR74 increased basal level of lipolysis on human mature white adipocytes, while NPFF diminished the lipolytic effects elicited by NE [145]. Albeit the mechanism of GPR74 haplotype function has not been fully characterized, NPFF effects on lipolysis inhibition were corroborated in other studies, together with the evidence of a higher expression of GPR74 in samples from obese patients [146]. 

Collectively, of the numerous peptide GPCRs expressed in adipose tissues, only a small fraction was studied in more detail. Clearly, further studies are needed to better understand and target such GPCRs to ameliorate metabolic diseases and treat obesity.

## 10. Frizzled/Smoothened

Frizzled receptor signaling is rather complex, the activation of such GPCRs occur through canonical Wnt/β-catenin, non-canonical planar cell polarity, and Wnt/Ca2+ pathways [147]. Only recently, the role of classical G protein signaling has been extended to such GPCRs [148]. The multifaceted signaling and the lack of selective pharmacological tools have hampered the study of their role in adipocytes and lipolysis. A role for the Wnt/β-catenin signaling as regulator of mesenchymal cell fate determination, promoting osteoblastogenesis and inhibiting adipogenesis was described [149]. Specifically, loss of β-catenin in adipocytes resulted in down-regulation of many genes involved in the de novo lipogenesis pathway, and knockout of β-catenin in adipocytes leads to a smaller proportion of monounsaturated fatty acid species compared to control adipocytes, altogether indicating the importance of the system for adipogenesis regulation. Mice lacking β-catenin displayed a decreased level of circulating triacylglycerols [149]. Further evidence showed how hedgehog/smoothened signaling is not only a determinant of the white versus brown cell fate [150], but also promotes lipolysis in adipose tissue by directly regulating Bmm/ATGL lipase [151]. Finally, Wnt signaling was reported to be involved in the regulation of lipolysis in human abdominal subcutaneous adipocytes [152].

## 11. Adhesion and GPCRs Activated by Tethered Agonists

Adhesion GPCRs (aGPCRs) share a common seven-transmembrane general architecture with class A, rhodopsin-like GPCRs. On the extracellular side, the amino-terminal domain is rather big when compared to most other GPCRs. The extracellular portion harbors an autocatalytic domain (GPCR autoproteolysis-inducing (GAIN) domain) where a GPCR proteolysis site (GPS) dissociate, upon self-cleavage in two components [153]. The N-terminal fragment (NTF) composed of adhesion motifs is liberated, and the C-terminal fragment (CTF) is then capable of intracellular signaling due to the activation by the tethered fragment agonist (a.k.a. Stachel peptide) [153]. Despite evidence of high aGPCRs expression in the adipose tissues, little is known regarding aGPCRs and adipose tissue lipolysis. For instance, exogenous aGPCR ADGRG2/GPR64 (Gs > Gq)-specific stachel peptide administration elicited robust glycerol release in 3T3-L1 cells and primary white adipocytes [154]. Mice lacking the aGPCR ADGRF5/GPR116 (Gq) displayed more pronounced FFA and triglyceride levels upon high-fat diet, thus suggesting a role for this aGPCR in controlling lipolysis [155]. 

GPR3 is not described as adhesion GPCR, its signaling, however, is activated by its N-terminal domain, leading in turn to an increase in cAMP levels [156]. This constitutive activity is, in BAT, important for lipolysis and thermogenesis. The expression of GPR3 is finely regulated and induced during cold exposure [156].

Taken together, aGPCRs clearly offer a potential for modulating lipolysis in the future. However, the development of novel pharmacological agents, capable of activating or inhibiting aGPCRs, is required to fully clarify the potential of these GPCRs. 

## 12. Olfactory and Opsin

Olfactory GPCRs are mainly known for their role in the olfactory epithelium, where they are the main odorant sensors capable of communicating volatile molecules presence and amount to the olfactory bulb and the brain [157]. Nevertheless, their presence has been identified in other tissues and therefore been named ectopic olfactory receptors (eORs) [157]. Among the tissues and organs, especially Olfr544 recently received attention [158]. For instance, Olfr544 is activated by azelaic acid (AzA): AzA increased the levels of cAMP in 3T3-L1 cells with concomitant release of glycerol [158]. Intriguingly, in vivo, the acute AzA injection induced lipolysis in wild-type mice and this effect was abrogated in Olfr544 knockout mice [158]. 

Even more puzzling is the role of photoreceptive non-visual opsins that are expressed in tissues outside the eye, mostly the brain and testis. Anatomical profiling of GPCRs reveals that Opn3 transcripts are highly expressed in adipose tissues [159]. Brown adipocytes lacking the Opn3 gene displayed a diminished glycerol release compared to wild type in basal conditions and when stimulated with the beta3 adrenergic receptor agonist CL-316243 or the phosphodiesterase inhibitor IBMX [160]. This diminished response was likely due to a reduced level of the ATGL. The use of light on wild-type brown adipocytes did not alter lipolytic pathways or lipolysis per se, however it increased both basal and maximal VO2 when palmitate was provided as fuel [160]. 

GPCRs coupling, molecular targets, and involvement in lipolysis are summarized in Table 1.

## 13. Conclusions

Because obesity is becoming endemic in Western countries, the development of novel approaches to diminish body mass indexes is needed. With the present review, we sum up the relevant literature and trends pointing towards the regulation of lipolysis via GPCR. GPCRs, accounting for a third of all marketed drugs and are thus an intriguing option, also for drug repurposing. 

So far, the main focus of adipocyte-centered drug discovery approaches was NE and the β3 adrenergic receptor. Mirabegron is a beta 3 adrenergic receptor-selective agonist [161], which has been approved to treat overactive bladder. Its clinical administration improved oral glucose tolerance and insulin sensitivity [162]. While in subcutaneous WAT, mirabegron treatment stimulated lipolysis, reduced fibrotic gene expression, and increased alternatively activated macrophages [162]. These effects have been linked to the amplitude of browning occurring in the WAT [162]. Similar findings indicated that human BAT metabolic activity could be increased after chronic pharmacological stimulation with mirabegron and support the investigation of β3-AR agonists as a treatment for metabolic disease [163]. However, the role of β3-AR in human brown and beige fat has recently been challenged [58]. Moreover, a broad spectrum of other GPCRs have been identified in different adipose tissue depots and cell types. Therefore, we review here multiple GPCRs involved in the regulation of lipolysis and of lipid content of the AT. 

Importantly, BAT stimulation using adrenergic agonists is accompanied by significant cardiovascular side-effects [54]. Recent results regarding the effects of an increase in inosine concentration recommend the ENT1/inosine as an innovative paradigm for future anti-obesity therapies [61].

Regarding the application of purines and small molecules capable of targeting the Ado receptors, RPR749 and its methylated metabolite are orally active and selective adenosine A1R agonists that can inhibit lipolysis and lower plasma triglyceride levels in a variety of animal models. RPR749 also appears to lower FFA and insulin levels and may have additional lipid-modifying effects. 

A clinical study evaluated the safety, pharmacokinetics, and pharmacodynamics (effect on FFA) after a single oral dose of up to 200 mg RPR749 or placebo. RPR749 can reduce circulating levels of FFA that can be related to plasma RPR749 concentrations and thus possesses pharmacological properties that may be beneficial in treating hyperlipidemia [164]. 

Collectively, lipolysis is a very important physiologic mechanism, central to the release of energy, in the form of FFAs from the WAT, and in the form of heat from the BAT. However, the liberation of FFAs from AT to other organs, including the liver, might be problematic. Thus, to harness the energy-dissipating function of BAT, it would be necessary to find BAT-specific regulators/ligands of lipolysis in order to avoid flooding of the body with FFA released from WAT. If BAT-specific activation of lipolysis (and energy expenditure) could be achieved, lipolysis may be certainly a target for the treatment of metabolic diseases and obesity. 

Very importantly, the increase in lipolysis in BAT would increase metabolic health, as described by Becher and colleagues [165]. A better knowledge of the GPCRs capable of modulating lipolysis and specifically expressed in different adipose tissues is required for novel therapeutic approaches to treat obesity and metabolic syndromes. 

## Figures and Tables

**Table 1:** Effects of major GPCRs on lipolysis.

GPCR	IUPHAR Name	Coupling	Molecular Targets ^a^	Lipolysis ^b^
beta 1 adrenergic	β1-adrenoceptor	Gs > Gi	AC, PKA, GC	+
beta 2 adrenergic	β2-adrenoceptor	Gs > Gi	AC, PKA, GC	+
beta 3 adrenergic	β3-adrenoceptor	Gs > Gi	AC, PKA, HSL, GC	+
A1R	A1 receptor	Gi > Gs, Gq	AC, PLC	−
A2AR	A2A receptor	Gs > Gq	AC, PLC	+
A2BR	A2B receptor	Gs > Gq	AC, PLC	+
D1	D1 receptor	Gs	AC, PLC	−
D2	D2 receptor	Gi	AC	− (+humans)
5-HT2A	5-HT2A receptor	Gq > Gi	PLC, AC	−
FFAR4/GPR120	FFA4 receptor	Gq	PLC	+ (?)
FFAR1/GPR40	FFA1 receptor	Gq > Gs	AC, PLC, PLA2	+
CB1	CB1 receptor	Gi > Gs	AC	−
GPER	GPER	Gi > Gs	PLC, AC	+
GprC6A	GPRC6 receptor	Gq	PLC	+ (?)
GPR183/EBI2	GPR183	Gi	AC	−
ETA	ETA receptor	Gq	PLC, PLA2, PLD	+
CMKLR1	Chemerin receptor 1	Gi	AC	+/−
Apelin	Apelin receptor	Gi	AC, PKC	−
NPY1	Y1 receptor	Gi	AC	−
GPR74	NPPF2	Gi (?)	AC	−
Gpr64	ADGRG2	Gs > Gq	AC, PLC	+
Gpr3	GPR3	Gs	AC	+
Olfr544	none	Golf/Gs	?	+
Opn3	OPN3	Gs (?)	?	+

^a^ most known molecular targets; if unknown, not specific for adipocytes. Note: β-arrestins coupling was not indicated. ^b^ + stimulatory effects, − inhibitory effects. ? not well defined. AC: Adenylate cyclase; PKA: protein kinase A; GC: guanylate cyclase; HSL: hormone sensitive lipase; PLC: phospholipase C; PLA2: phospholipase A2; PLD, phospholipase D; PKC: protein kinase C.

## Data Availability

Data sharing not applicable.
